# Precision in long-term language evaluation after awake brain tumor surgery

**DOI:** 10.1093/nop/npaf090

**Published:** 2025-09-06

**Authors:** Barbara Vogrincic, Saskia Mooijman, Arnaud Vincent, Bram Bulté, Elke De Witte, Evy Visch-Brink, Djaina Satoer

**Affiliations:** Faculty of Medicine, University of Ljubljana, Slovenia; Erasmus MC, University Medical Center Rotterdam, Department of Neurosurgery, The Netherlands; Centre for Language Studies, Radboud University, Nijmegen, the Netherlands; Erasmus MC, University Medical Center Rotterdam, Department of Neurosurgery, The Netherlands; Erasmus MC, University Medical Center Rotterdam, Department of Neurosurgery, The Netherlands; Brussels Centre for Language Studies, Vrije Universiteit Brussel, Belgium; Erasmus MC, University Medical Center Rotterdam, Department of Neurosurgery, The Netherlands; Erasmus MC, University Medical Center Rotterdam, Department of Neurosurgery, The Netherlands; Erasmus MC, University Medical Center Rotterdam, Department of Neurosurgery, The Netherlands

**Keywords:** awake craniotomy, Diagnostic Instrument for Mild Aphasia (DIMA), gliomas, long-term language outcome, language assessment

## Abstract

**Background:**

Language deficits caused by gliomas can persist beyond the short term, and should not be overlooked due to their influence on a patient’s quality of life. The aim of this study was to investigate language impairments and long-term outcome after awake surgery.

**Methods:**

The Diagnostic Instrument for Mild Aphasia (DIMA) and traditional language tests were administered preoperatively (T1; *n* = 79), and 3 (T2; n = 68) and 12 months (T3; *n* = 35) postoperatively in the groups of patients with less (Group 1) and more (Group 2) invasive brain tumors.

**Results:**

Preoperative deficits were found with DIMA at the level of lexico-syntax in Group 1 in both hemispheres and at the level of phonology and lexico-syntax in Group 2. With traditional language tests, preoperative deficits were detected at the level of word retrieval and verbal fluency in all groups. Only DIMA revealed long-term mild language impairments, which were observed at the phonological and semantic level in patients with left-hemispheric tumors, and at the lexico-syntactic level in those with right-hemispheric tumors.

**Conclusions:**

In this cohort, mild language impairments were noted long-term with DIMA across different linguistic levels, irrespective of hemispheric tumor location. Alongside the traditional language tests, DIMA represents a valuable addition for the diagnostic evaluation of language impairments in patients with gliomas.

## Graphical Abstract

**Graphical Abstract:**
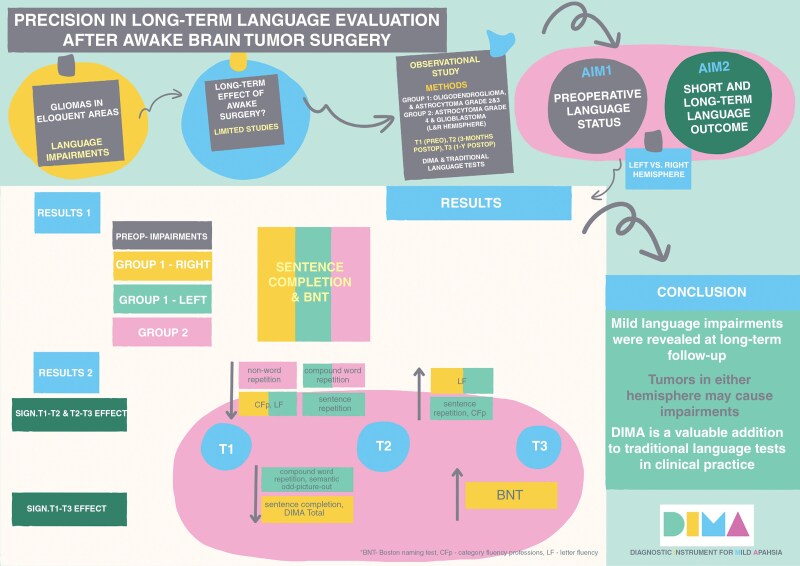

**Key Points:**

- Mild language impairments were revealed at long-term follow-up.
- Gliomas in both hemispheres may cause impairments.
- DIMA is a valuable addition to traditional language tests in clinical practice.

**Importance of the Study**

Since aphasia is one of the factors that can have an impact on the quality of life of a patient with a glioma, not only short-term but especially long-term language impairments should not be overlooked. In this cohort, preoperative deficits were found with DIMA at the level of lexico-syntax in patients with less invasive tumors in both hemispheres and at the level of phonology and lexico-syntax in patients with more invasive tumors. With traditional language tests, preoperative deficits were detected at the level of word retrieval and verbal fluency. Only DIMA revealed long-term mild language impairments, which were observed in the repetition and semantic odd-picture-out subtests in patients with left-hemispheric tumors, and sentence completion and DIMA Total in those with right-hemispheric tumors. We therefore suggest adding DIMA to standard clinical practice for better language diagnosis and patient counseling on outcome.

Gliomas are often located in eloquent brain areas, hence, patients often suffer from cognitive impairments.^1^ The language domain is frequently affected, and it is therefore critical to assess language abilities in this patient group.^2^ At the group level, patients with gliomas most notably suffer from deficits in verbal fluency and object naming.^3^ At the individual level, the observed incidence of language impairments varies widely and is reported to range from 3% to 61%.^4–7^ Variability can be due to differences between diagnostic tests, patient’s age, stage of the treatment and disease as well as tumor variables such as WHO grade, molecular characteristics (IDH1, IDH2, IDH-wildtype, 1p/19q), size, and location (hemisphere, lobe).^1,5,8–10^

Awake surgery with direct electric stimulation (DES) is currently considered the gold standard treatment for slow growing gliomas, as it allows for maximal tumor resection whilst preserving cognitive functions.^11,12^ Even in a subgroup of glioblastoma patients the awake procedure has been found to be feasible and safe.^13^ Yet, despite careful preoperative planning and intraoperative monitoring, surgery may lead to postoperative decline of language functions. The general pattern appears to be a deterioration in language function immediately postsurgery, which may improve to the preoperative baseline in the months that follow.^6,14–16^ However, most research investigating postoperative language deficits focused on a relatively short follow-up (i.e. <3 months) and, as such, the precise long-term recovery trajectory has not yet been established.^16^

Several recent studies have emphasized the importance of long-term follow-up assessments, given the persistence of language impairments over time.^3,17–20^ For example, it was found that naming and letter fluency abilities recovered at 1-year postsurgery, while category fluency remained impaired.^3^ Another study also found persisting language disorders postoperatively, as 69% of patients with grade 2 and 89% of patients with grades 3 and 4 showed impairments.^18^ As previously mentioned, language impairments may also vary due to glioma grade. In particular, language impairments of patients with gliomas grade 2 are often more subtle.^21^ At the same time, traditional language tests often focus only on a limited range of language domains or on overall language scores, ignoring many specific language functions.^18^ The result is that certain impairments cannot be objectively established, even though patients report experiencing language difficulties.^18,22^ Mild language impairments can have profound effects on a patient’s quality of life, for example at a professional (returning to work, performing work related tasks, functioning in a busy environment) and personal level (e.g. day-to-day communication with family, reading movie subtitles, daily chores). These difficulties can lead to frustration, lack of self-confidence and can hinder employability and career.^23,24^ Therefore, it is essential to have language tests that can objectively measure language impairments and that can target specific language domains or levels.^16^

Above all, different linguistics levels such as syntax, semantics, phonology, morphology, pragmatics and their integration are required for successful communication.^25^ Our research group addressed these challenges by investigating the spontaneous speech abilities of patients with gliomas and found that these analyses were sensitive to detect impairments, as demonstrated by the occurrence of more incomplete sentences and shorter utterances in patients’ speech samples compared to healthy speakers.^20^ However, analyzing spontaneous speech is time-consuming and not always feasible in all settings of clinical practice.

The exact prevalence of language impairments and the extent of recovery, especially in the later stages after awake surgery in patients with gliomas, remains uncertain due to a lack of studies with sufficiently fine-grained language tests in large patient groups. Recently, the Diagnostic Instrument for Mild Aphasia (DIMA) was developed to provide a diagnostic tool for mild language disturbances,^26^ which may occur in patients with gliomas. The DIMA is a standardized test that evaluates language abilities at all linguistic levels, but it has not yet been administered to a larger group of patients with gliomas. The aim of the present study is to establish whether patients with gliomas suffer from preoperative language impairments, measured with the DIMA and traditional language tests, and to investigate the short- and long-term effects of awake surgery on language outcome.

## Materials and Methods

### Study Design

This single-center, longitudinal observational cohort study followed the standard clinical protocol of the Erasmus MC - University Medical Center, Rotterdam, the Netherlands, for awake brain surgery. Language tests were administered at three time points: shortly before surgery (T1), 3 months postsurgery (T2), and 1-year postsurgery (T3).

### Setting, Clinical Protocol, and Participants

Eligible patients were Dutch-speaking adults (≥18 years) who underwent awake glioma surgery at the Erasmus MC - University Medical Center, Rotterdam, The Netherlands between March 2015 and August 2021. Patients gave their written informed consent prior to participation, and all data were collected with the permission of the Medical Ethical Committee of the Erasmus MC (MEC-2013-090-A-0001).

After craniotomy and durotomy, the patients underwent conventional DES. While the neurosurgeon performed cortical and subcortical stimulation with a bipolar electrode, the clinical linguist carried out intraoperative language assessment using specific tasks from the Dutch Intraoperative Linguistic Protocol (DuLIP)^27^ based on tumor location and preoperative language level. (A clinical linguist typically focuses on language diagnostics, while a speech and language pathologist covers both diagnostics and therapy. This distinction may vary across countries. At the Erasmus MC, awake language monitoring is carried out by both professionals.) If an eloquent area during stimulation was found, it was labeled with a sterile number tag. During the resection phase, language and speech were monitored by means of additional DuLIP tasks and spontaneous speech. Notably, in our center all patients with gliomas receive dexamethasone (8 mg p/d) as standard care, which starts at the latest 1 day before surgery, with a phase-out schedule of maximally 10 days.

Patients were included in further statistical analyses if postoperative pathological examination confirmed a glioma as an integrated diagnosis and if they completed the DIMA as part of their standard clinical workup (*n* = 105). We subsequently excluded 3 patients, as they were confirmed as nongliomas on histopathology. In this sample, we used the fifth edition of the WHO classification of Tumors of the Central Nervous System (WHO CNS5). The WHO CNS5 of adult-type gliomas classification includes 3 types of gliomas: (i) Astrocytoma, IDH-mutant, (ii) Oligodendroglioma, IDH-mutant and 1p/19q-codeleted, and (iii) Glioblastoma (GBM), IDH-wildtype.^10^ Patients were subdivided into 2 groups based on the gliomas’ malignancy: a group with lower malignancy or less invasive gliomas (Group 1) and a group with high malignancy or more invasive gliomas (Group 2). Group 1 contains patients with astrocytoma, IDH-mutant grades 2 and 3 and oligodendroglioma, IDH-mutant and 1p/19q-codeleted grades 2 and 3. Group 2 includes patients with astrocytoma, IDH-mutant grade 4 and GBM, IDH-wildtype. Additionally, we excluded patients who had undergone tumor re-resection (*n* = 23). MRI was carried out as part of the clinical protocol of patients with gliomas and was used to determine the brain tumor’s location.

### Materials

The DIMA was developed to detect mild aphasia in brain tumor patients and other neurological populations.^26^ It was standardized on a representative sample of healthy, adult native Dutch speakers. The DIMA measures language production across different linguistic levels: phonological, semantic and lexico-syntax (see Supplementary material S1 for additional information about DIMA).^26^ (DIMA also includes sentence judgment tests, but these were only administered to a subgroup of patients and are therefore not discussed in the present article.^22^)

The traditional language tests administered to the patients were the Boston Naming Test (BNT^28^) for word retrieval abilities, the shortened Token Test (sTT^29^) to measure comprehension and the presence and severity of aphasia, and verbal fluency (VF) tests, i.e. Category (Animals [CFa] and Professions [CFp]^30^) as well as Letter Fluency (LF^31^) to assess the flexibility of lexical retrieval.

### Procedure

A clinical linguist assessed patients individually in the hospital’s outpatient clinic as part of the standard preoperative and postoperative procedures. The same clinical linguist tested the patients intraoperatively. However, intraoperative data are not part of this study. Instructions for each (pre- and postoperative) test were given before administration. The sessions were audio-recorded to verify administration. The entire appointment, which included an interview and a standard neurolinguistic test battery (including nonlinguistic cognitive tests, which are outside the scope of the present study), generally lasted around 90 min.

### Analysis

Data were analyzed using the statistical programming language R, version 4.1, in RStudio version 2021.09.1 2 (R Core Team, 2020). We used the following packages: “lme4,” “lmerTest,” “emmeans,” “tidyverse,” “ggplot2” and “rstatix.”^32–37^

To enable hemisphere-specific analysis, we further subdivided the patients in Group 1 according to tumor lateralization. Statistical analyses were then performed separately for each subgroup. Given the limited sample size of Group 2, this group was not subdivided by hemisphere to preserve statistical power.

To determine whether patients were impaired on the tests, raw scores were compared to normative data.^28–31^ Published DIMA normative data,^26^ which served as the control group in this study, accounted for age (2 age groups, <55 years ≤) and education (2 education groups, ≤12 year <). *Clinical* (mild) impairments were operationalized as *z-*scores of ≤−1.5 (traditional language tests) or <7th percentile (DIMA), corrected for age, years of education, or both. For the traditional language tests, we only report the percentage of impaired patients at each time point, as we did not have access to the raw norm data. For DIMA, we used Fisher’s exact test to compare the proportion of patients with deviant scores on DIMA preoperatively to the norm group and calculated Cramer’s V as a measure of effect size. As a general rule of thumb, Cramer’s V values around 0.1 point to a small effect, around 0.3 to a medium effect, and 0.5 to a large effect. Pairwise comparisons were conducted for each time point, and the Benjamini–Hochberg procedure was used to correct for multiple testing.

To establish the effects of surgery over time, we ran a series of linear mixed-effects regressions, with patient *z*-scores as outcome variable. As the analyses for Group 1 were split up according to hemispheric tumor location, time point was the only fixed effect in the corresponding models. For Group 2, both time point and laterality of the tumor location constituted the fixed effects. There was not enough data to model interactions. Patients were included in the models as random intercepts to account for by-participant variation. Since we used *z*-scores as outcomes, the model coefficients show estimated differences in terms of standard deviation (which can be considered a type of effect size).

## Results

### Participants

Table 1 provides demographic and tumor-related information for the 79 participants included at baseline testing, divided into groups. Group 1 consisted of 54 patients, while Group 2 comprised 25 patients. Most withdrawals at T2 and T3 were linked to clinical deterioration (in Group 2, 7 patients deceased within 1 year and in 11 patients the disease showed progression). The attrition rate between T1 and T2 was 14% and 47% between T2 and T3 (see Supplementary Table S2). At the Erasmus MC, patients with GBM do not routinely receive standard neurolinguistic follow-up 1-year postsurgery, which further contributed to the high attrition rate in Group 2.

**Table 1.** Demographic and clinical information of the patient group at T1

Variable			*n*	Percentage
Mean age at T1 (range)	43.3 (21.3-73.3)			
Mean education (range)	14 (8.0-23.0)			
Groups				
	Group 1		54	68
	Group 2		25	32
Sex				
	Female	Group 1	19	24
		Group 2	8	10
	Male	Group 1	35	44
		Group 2	17	22
Handedness				
	Left	Group 1	7	8
		Group 2	2	3
	Right	Group 1	47	60
		Group 2	23	29
Group 1 and Gorup 2 Tumor type				
	Oligodendroglioma, IDH-mutant, and 1p/19q-codeleted, WHO grade 2,3		31	39
	Astrocytoma, IDH-mutant, WHO grade 2,3		23	29
	Astrocytoma, IDH-mutant, WHO grade 4		4	5
	Glioblastoma, IDH wild type grade 4		21	27
Hemisphere				
	Left	Group 1	27	34
		Group 2	18	23
	Right	Group 1	27	34
		Group 2	7	9
Lobe				
	Frontal	Group 1	33	42
		Group 2	6	7
	Temporal	Group 1	9	11
		Group 2	6	8
	Parietal	Group 1	8	10
		Group 2	6	8
	Fronto-parietal	Group 1	2	3
		Group 2	1	1
	Temporo-parietal	Group 1	0	0
		Group 2	3	4
	Fronto-temporal	Group 1	1	1
		Group 2	1	1
	Frontal/insula	Group 1	1	1
		Group 2	1	1
	Parieto-occipital	Group 1	0	0
		Group 2	2	2
Grade				
	2		46	58
	3		8	10
	4		25	32
Extent of resection^a^				
	Gross total resection	Group 1	16	20
		Group 2	6	8
	Subtotal resection	Group 1	29	37
		Group 2	17	21
	Partial resection	Group 1	9	11
		Group 2	2	3
Adjuvant therapy within 1 year postsurgery				
	Radiotherapy	Group 1	0	0
		Group 2	2	2
	Chemotherapy	Group 1	3	4
		Group 2	7	9
	Chemo- and radiotherapy	Group 1	26	33
		Group 2	12	15
	No therapy	Group 1	25	32
		Group 2	4	5
Progression free survival within one year				
	Group 1		50	63
	Group 2		10	13

^a^Gross total resection is defined as the removal of all tumors; subtotal resection is defined as the minimal residue of a glioma, and partial resection is defined as 50% of the residue of a glioma, as gauged by magnetic resonance imaging.

### Preoperative Language Impairments

**Figure 1.**
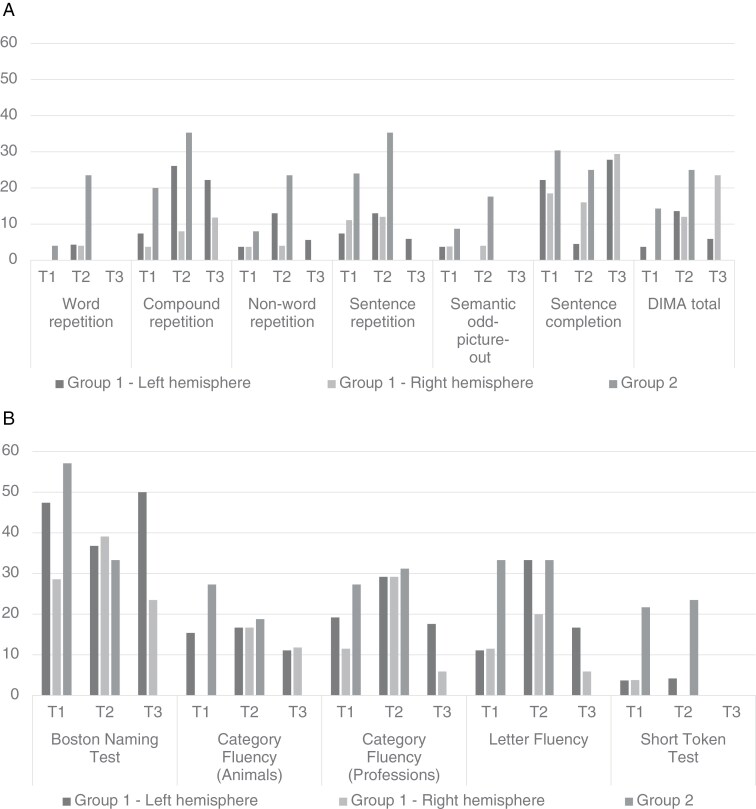
(A) Percentage of clinical (mild) language impairments on the DIMA subtests and DIMA Total. (B) Percentage of clinical (mild) language impairments on the traditional language tests.

Figure 1A and B show the percentage of clinical impairments for each subgroup (at 3 time points) for DIMA and the traditional language tests. Exact figures can be consulted in Supplementary Table S3. Supplementary Table S4 contains the results of the associated Fisher’s exact tests for DIMA.

#### Group 1 (less invasive tumors).

—For the DIMA, clinical preoperative impairments (T1) were found in 3.7% (nonword repetition, semantic odd-picture-out and DIMA Total) to 22.2% (sentence completion) of patients with a tumor in the left hemisphere (LH), and 3.7% (compound word repetition and nonword repetition) to 18.5% (sentence completion) of patients with a tumor in the right hemisphere (RH). Compared with the published norms,^26^ both LH and RH patients showed a significantly higher incidence of clinical impairments preoperatively for one DIMA subtest: sentence completion (LH: *P* = 0.004, Cramer’s *V* = 0.235, RH: *P* *=* 0.018, Cramer’s *V* = 0.193). As to the traditional language tests, the BNT had the highest percentage of clinical impairments in both LH (47.4%) and RH patients (28.6%) before surgery.

#### Group 2 (more invasive tumors).

—Clinical preoperative impairments were found in the DIMA in 4% (word repetition) to 30.4% (sentence completion) of patients with more invasive gliomas (Group 2). Compared to the published norms,^26^ patients showed a significantly higher incidence of clinical impairments for compound word repetition (*P* = 0.022, Cramer’s *V* = 0.171), sentence repetition (*P* = 0.002, Cramer’s *V* = 0.250) and sentence completion (*P* < 0.001, Cramer’s *V* = 0.309). When considering the traditional language tests in Group 2, the BNT had the highest percentage of clinical impairments preoperatively (57.1%).

### Recovery After Awake Craniotomy

**Figure 2:**
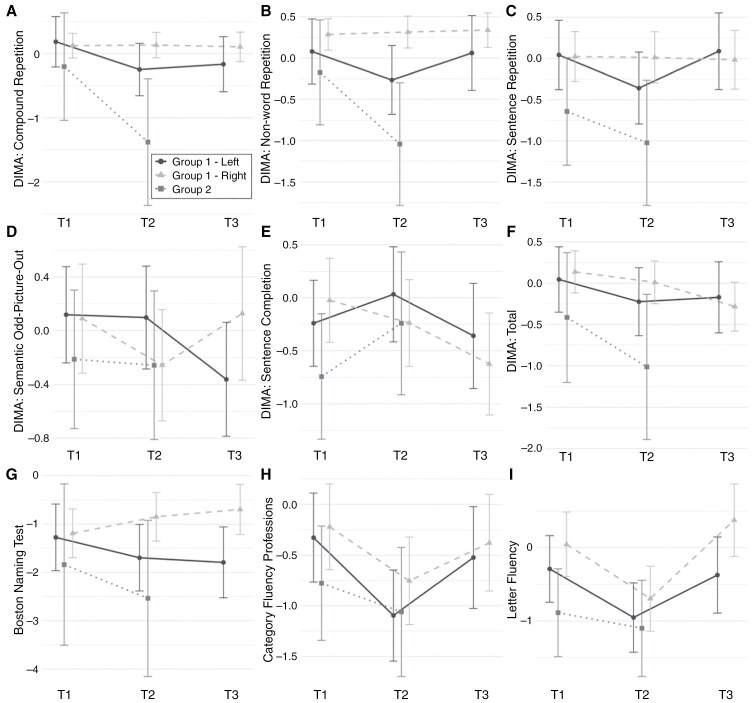
Effect plots for regression models with at least one significant time effect across patient groups. (A) A decline between T1-T2 and T1-T3 in G1-LH and a decline between T1 and T2 in G2. (B) A decline between T1 and T2 in G2. (C) A decline between T1 and T2 and an improvement between T2 and T3 in G1-LH. (D) A decline between T1 and T3 in LH-G1. (E) A decline between T1 and T3 G1-RH. (F) A decline between T1 and T3 in G1-RH. (G) An improvement between T1-T2 and T1-T3 in G1-RH. (H) A decline between T1-T2 and an improvement between T2-T3 in G1-LH and a decline between T1-T2 in G1-RH. (I) A decline between T1-T2 and an improvement between T2-T3 in G1-LH and G1-RH. *G1, Group 1; G2, Group 2; LH, left-hemispheric tumor patients; RH, right-hemispheric tumor patients. The legend is valid for each plot.

For a more detailed breakdown of percentages of clinical impairments for each subgroup at T2 and T3, please refer to Supplementary Table S3 and corresponding visualizations in Figure 1A and B for DIMA and for the traditional language tests. In this section we present the results of the mixed-effects regression analyses used to model the evolution of test scores over time. All model estimates are provided in Tables 2-. To enhance clarity, Figure 2 visualizes the model outcomes by providing effect plots for all tests for which at least one significant time effect was found.

**Table 2.** Outcomes linear regression models for left-hemispheric tumor patients in Group 1^a^

	Estimate	Std. error	df	*t-*value	*P-*value
**DIMA: Word repetition**					
Intercept	0.00	0.17	65.00	0.027	0.978
T1 vs. T2	0.26	0.23	65.00	1.126	0.264
T2 vs. T3	0.01	0.26	65.00	0.023	0.982
T1 vs. T3	−0.25	0.25	65.00	−1.026	0.309
**DIMA: Compound word repetition**					
Intercept	−0.25	0.20	39.60	−1.22	0.230
T1 vs. T2	**0.43**	**0.15**	**40.93**	**2.86**	**0.007**
T2 vs. T3	0.08	0.17	40.84	0.49	0.627
T1 vs. T3	−**0.35**	**0.17**	**41.45**	−**2.10**	**0.042**
**DIMA: Nonword repetition**					
Intercept	−0.27	0.21	48.46	−1.27	0.209
T1 vs. T2	0.34	0.20	39.96	1.71	0.010
T2 vs. T3	0.33	0.23	40.18	1.45	0.156
T1 vs. T3	−0.02	0.22	41.13	-0.08	0.936
**DIMA: Sentence repetition**					
Intercept	−0.36	0.22	37.48	-1.65	0.108
T1 vs. T2	**0.40**	**0.16**	**38.47**	**2.53**	**0.016**
T2 vs. T3	**0.45**	**0.18**	**38.52**	**2.45**	**0.019**
T1 vs. T3	0.05	0.17	39.06	0.26	0.780
**DIMA: Semantic odd-picture-out**					
Intercept	0.10	0.19	56.67	0.51	0.613
T1 vs. T2	0.02	0.21	42.65	0.10	0.921
T2 vs. T3	−0.46	0.24	43.32	−1.95	0.057
T1 vs. T3	−**0.48**	**0.23**	**44.35**	−**2.10**	**0.041**
**DIMA: Sentence completion**					
Intercept	0.03	0.22	63.42	0.15	0.885
T1 vs. T2	−0.27	0.29	30.01	−0.93	0.358
T2 vs. T3	−0.39	0.33	33.08	−1.20	0.237
T1 vs. T3	−0.12	0.31	33.22	−0.38	0.706
**DIMA total**					
Intercept	−0.22	0.21	35.55	-1.09	0.282
T1 vs. T2	0.26	0.14	36.90	1.95	0.059
T2 vs. T3	0.05	0.16	37.02	0.34	0.739
T1 vs. T3	−0.21	0.15	37.30	−1.41	0.166
**Boston naming test**					
Intercept	−1.69	0.34	41.46	−4.93	<0.001
T1 vs. T2	0.42	0.37	34.17	1.15	0.257
T2 vs. T3	−0.10	0.37	32.14	−0.26	0.798
T1 vs. T3	−0.52	0.38	33.17	−1.37	0.181
**Category fluency animals**					
Intercept	−0.45	0.22	37.57	−2.02	0.050
T1 vs. T2	−0.01	0.18	39.93	−0.03	0.976
T2 vs. T3	0.23	0.20	40.75	1.17	0.251
T1 vs. T3	0.24	0.20	40.82	1.21	0.232
**Category fluency professions**					
Intercept	−1.10	0.23	44.93	−4.87	<0.001
T1 vs. T2	**0.77**	**0.21**	**40.45**	**3.67**	**<0.001**
T2 vs. T3	**0.57**	**0.24**	**41.99**	**2.37**	**0.023**
T1 vs. T3	−0.20	0.24	42.01	−0.83.	0.413
**Letter fluency**					
Intercept	0.95	0.24	47.82	−4.02	<0.001
T1 vs. T2	**0.66**	**0.22**	**41.87**	**2.97**	**0.005**
T2 vs. T3	**0.58**	**0.25**	**42.74**	**2.29**	**0.027**
T1 vs. T3	−0.08	0.25	43.19	−0.34	0.738
**Shortened token test**					
Intercept	0.25	0.23	29.53	1.20	0.281
T1 vs. T2	−0.20	0.10	38.97	−1.96	0.058
T2 vs. T3	−0.15	0.12	39.09	−1.25	0.218
T1 vs. T3	−0.05	0.12	39.21	0.44	0.664

^a^Statistically significant parameters are indicated in bold.

**Table 3.** Outcomes linear regression models for RH tumor patients in Group 1^a^

	Estimate	Std. Error	df	*t-*value	*P*-value
**DIMA: Word repetition**					
Intercept	0.02	0.13	65.98	0.15	0.884
T1 vs. T2	0.26	0.17	46.63	1.51	0.139
T2 vs. T3	0.27	0.20	52.20	1.35	0.182
T1 vs. T3	0.01	0.20	51.90	0.03	0.979
**DIMA: Compound word repetition**					
Intercept	0.13	0.10	49.73	1.33	0.188
T1 vs. T2	−0.01	0.10	37.79	−0.09	0.931
T2 vs. T3	−0.30	0.12	40.50	−0.23	0.821
T1 vs. T3	−0.02	0.12	40.44	−0.16	0.878
**DIMA: Non-word repetition**					
Intercept	0.31	0.10	35.50	3.25	0.003
T1 vs. T2	−0.03	0.07	39.67	−0.42	0.674
T2 vs. T3	0.03	0.08	40.54	0.29	0.773
T1 vs. T3	0.05	0.09	40.57	0.66	0.513
**DIMA: Sentence repetition**					
Intercept	0.01	0.15	46.43	0.08	0.934
T1 vs. T2	0.01	0.15	38.14	0.07	0.946
T2 vs. T3	−0.03	0.18	40.38	−0.16	0.871
T1 vs. T3	−0.04	0.18	40.35	−0.23	0.823
**DIMA: Semantic odd-picture-out**					
Intercept	−0.26	0.21	61.75	−1.24	0.220
T1 vs. T2	0.35	0.26	41.08	1.33	0.191
T2 vs. T3	0.39	0.30	45.09	1.29	0.206
T1 vs. T3	0.04	0.29	44.28	0.13	0.899
**DIMA: Sentence completion**					
Intercept	−0.24	0.21	54.85	−1.17	0.248
T1 vs. T2	0.22	0.22	40.45	0.95	0.347
T2 vs. T3	−0.39	0.26	43.53	−1.47	0.149
T1 vs. T3	−**0.60**	**0.26**	**43.43**	−**2.32**	**0.025**
**DIMA total**					
Intercept	0.01	0.13	45.68	0.08	0.938
T1 vs. T2	0.13	0.13	38.34	0.99	0.328
T2 vs. T3	−0.29	0.15	40.10	−1.99	0.053
T1 vs. T3	−**0.42**	**0.15**	**39.62**	−**2.88**	**0.006**
**Boston Naming test**					
Intercept	−0.85	0.25	30.00	−3.40	0.002
T1 vs. T2	−**0.34**	**0.14**	**32.04**	−**2.43**	**0.021**
T2 vs. T3	0.15	0.15	31.76	1.04	0.307
T1 vs. T3	**0.49**	**0.16**	**32.55**	**3.10**	**0.004**
**Category Fluency Animals**					
Intercept	−0.41	0.21	47.45	−1.94	0.059
T1 vs. T2	0.25	0.21	40.30	1.20	0.239
T2 vs. T3	0.27	0.24	41.14	1.14	0.259
T1 vs. T3	0.02	0.23	41.11	0.10	0.921
**Category fluency professions**					
Intercept	−0.75	0.22	42.25	−3.48	0.001
T1 vs. T2	**0.53**	**0.19**	**40.01**	**2.86**	**0.007**
T2 vs. T3	0.37	0.21	40.47	1.76	0.086
T1 vs. T3	−0.16	0.21	40.48	−0.75	0.455
**Letter fluency**					
Intercept	−0.69	0.22	39.72	−3.12	0.003
T1 vs. T2	**0.74**	**0.18**	**39.85**	**4.02**	**<0.001**
T2 vs. T3	**1.07**	**0.21**	**40.82**	**5.02**	**<0.001**
T1 vs. T3	0.33	0.21	40.52	1.57	0.124
**Shortened token test**					
Intercept	0.17	0.16	30.60	1.03	0.312
T1 vs. T2	−0.09	0.10	31.68	−0.84	0.405
T2 vs. T3	−0.08	0.16	31.78	−0.62	0.543
T1 vs. T3	0.01	0.12	31.90	0.08	0.939

^a^Statistically significant parameters are indicated in bold.

**Table 4.** Outcomes linear regression models for brain tumor patients in Group 2^a^

	Estimate	Std. Error	df	*t-*value	*P-*value
**DIMA: Word repetition**					
Intercept	−0.17	0.37	39.00	−0.47	0.644
T1 vs. T2	−0.75	0.53	39.00	−1.42	0.164
Right hemisphere	0.36	0.56	39.00	0.63	0.530
**DIMA: Compound word repetition**					
Intercept	−0.37	0.48	31.67	−0.78	0.444
T1 vs. T2	−**1.20**	**0.52**	**20.52**	−**2.32**	**0.031**
Right hemisphere	0.59	0.81	22.46	0.73	0.473
**DIMA: Nonword repetition**					
Intercept	−0.33	0.36	31.35	−0.92	0.366
T1 vs. T2	−**0.89**	**0.38**	**20.82**	−**2.36**	**0.028**
Right hemisphere	0.56	0.62	23.20	0.90	0.378
**DIMA: Sentence repetition**					
Intercept	−0.77	0.37	29.83	−2.04	0.050
T1 vs. T2	−0.40	0.38	19.30	−1.06	0.302
Right hemisphere	0.44	0.65	22.16	0.67	0.509
**DIMA: Odd-picture-out**					
Intercept	−0.28	0.30	24.24	−0.93	0.364
T1 vs. T2	−0.04	0.21	15.49	−0.21	0.836
Right hemisphere	0.23	0.54	20.43	0.42	0.677
**DIMA: Sentence completion**					
Intercept	−0.75	0.35	26.77	−2.17	0.039
T1 vs. T2	0.51	0.32	18.10	1.59	0.130
Right hemisphere	0.03	0.59	21.02	0.05	0.959
**DIMA total**					
Intercept	−0.53	0.46	23.52	−1.15	0.263
T1 vs. T2	−0.59	0.45	12.93	−1.30	0.215
Right hemisphere	0.32	0.74	14.82	0.43	0.676
**Boston naming test**					
Intercept	−2.05	0.86	24.88	−2.39	0.025
T1 vs. T2	−0.75	1.01	12.62	−0.75	0.469
Right hemisphere	1.31	1.63	18.19	0.81	0.431
**Category fluency animals**					
Intercept	−1.11	0.28	29.04	−3.96	<0.001
T1 vs. T2	0.14	0.32	18.00	0.44	0.664
Right hemisphere	**0.92**	**0.44**	**18.37**	**2.12**	**0.048**
**Category fluency professions**					
Intercept	−0.96	0.33	25.26	−2.95	0.007
T1 vs. T2	−0.30	0.31	16.56	−0.96	0.349
Right hemisphere	0.57	0.53	18.56	1.08	0.296
**Letter fluency**					
Intercept	−1.17	0.34	24.00	−3.43	0.002
T1 vs. T2	−0.21	0.27	15.27	−0.79	0.441
Right hemisphere	0.87	0.57	19.93	1.52	0.144
**Shortened token test**					
Intercept	−2.31	1.05	23.25	−2.21	0.038
T1 vs. T2	−0.05	0.58	15.14	−0.09	0.926
Right hemisphere	1.59	1.89	20.81	0.84	0.410

^a^Statistically significant parameters are indicated in bold.

#### Group 1.

—Regarding the DIMA, LH patients demonstrated a significant decline between T1 and T2 in compound word and sentence repetition. Conversely, RH patients did not demonstrate any significant change between T1 and T2 on DIMA. Concerning the traditional language tests, LH patients suffered a significant decline between T1 and T2 in CFp and LF. RH patients showed the same pattern for CFp and LF, but demonstrated significant improvement in BNT. Between T2 and T3, LH patients showed a significant improvement in sentence repetition, as well as on the traditional tests CFp and LF. RH patients only showed a significant improvement for the traditional test LF. With respect to the long-term (T1-T3) effects of awake surgery, there was a significant decline for compound word repetition and semantic odd-picture-out in the DIMA for LH patients. RH patients displayed long-term significant decline in sentence completion and DIMA Total, and significant improvement on the traditional test BNT.

#### Group 2.

—In relation to the DIMA, Group 2 patients demonstrated a significant decline between T1 and T2 in compound and nonword repetition. There was no significant decline compared to the preoperative baseline for any of the traditional tests. A hemispheric effect emerged exclusively on the traditional test CFa, with RH patients scoring significantly higher overall.

## Discussion

Previous studies have shown that patients diagnosed with gliomas are at high risk of developing language impairments, varying from mild to profound severity. Especially mild language changes can be overlooked.^38^ Although patients may subjectively recognize these changes, most diagnostic language tools are not able to fully identify their presence. In this study, we analyzed the prevalence and characteristics of language impairments in a group of patients with gliomas who underwent awake brain surgery. We evaluated language function preoperatively (T1), 3 months after surgery (T2) and 1-year post-surgery (T3) with traditional language tests and DIMA, which is a new standardized diagnostic tool for mild language impairments. Prior to this study, DIMA had not been administered in a sizeable group of patients with gliomas, especially not long-term postsurgery. To the best of our knowledge, a detailed assessment of language function and communication in patients with gliomas in this late phase after surgery remains under-researched.^17^

### Preoperative Language Impairments

Preoperatively, the percentage of patients showing clinical impairments was the highest on tests for single-word retrieval (BNT), followed by lexico-syntax (DIMA sentence completion), VF (category and letter), and phonology (DIMA sentence repetition). This study also confirmed that DIMA alongside BNT and VF, enables the detection of a broader range of (mild) language impairments in a group of patients with gliomas in comparison to a healthy population as an effect of a tumor.

Both patients’ groups with tumors in the LH and RH presented a significantly higher incidence of clinical impairments compared with published norms at the level of phonology and lexico-syntax preoperatively. Compromised lexico-syntactic abilities in patients with brain tumors were also observed in previous studies.^20,39^ Tests that assess these two levels in DIMA seem to facilitate the detection of impairments in language function as a consequence of the tumor.

More invasive, higher-graded tumors affected language more severely than less invasive, lower-graded ones. In the present study, GBMs and astrocytomas grade 4 (Group 2) affected all linguistic levels to a substantial degree. These results confirm previous studies comparing functional outcomes of patients with a less invasive and a more invasive tumor, stating that patients with a less invasive tumor show milder language impairments compared to those with higher-graded tumors.^21^ This can be explained by the fact that GBMs and astrocytomas grade 4 are fast-growing with larger mass effect, which can easily disrupt a language network and its function.^1,40^ The reverse pattern holds for less invasive gliomas in which typically no or mild language deficits are observed as a result of a slow-growing tumor inducing plasticity in the central nervous system.^11,41^ Furthermore, patients in Group 2 displayed a significantly higher incidence of clinical impairments compared with published norms at the level of phonology and lexico-syntax at T1.

### Effects of Surgery on Language

Awake surgery with DES is the gold standard for treating patients with gliomas to prevent irreversible neurological outcomes.^12^ Language, which is one of the most important contributors to quality of life,^23^ has been found to deteriorate immediately after surgery, but can subsequently recover to baseline level.^6,14–16,42^ We observed similar language function fluctuations in this study. Results on both the DIMA and the traditional language tests (BNT, sTT, VF) showed language impairments due to the presence of the tumor, which may be temporarily aggravated by surgery. Generally speaking, with some exceptions, which we will discuss below, impairments were higher shortly after surgery, whereas long-term postsurgery patients returned to the preoperative level.

#### Left vs. right hemisphere in less invasive tumors

—LH patients (Group 1) displayed a higher percentage of clinical impairments at the level of phonology shortly after surgery and 1 year postoperatively. These results corroborate previous research showing that phonological tests are sensitive to detect language impairments, especially in the case of damaged arcuate fasciculus, and are a valuable indicator of language prognosis^43,44^ Short-term postoperatively (T1-T2), phonology (repetition) as well as VF (both category and letter) significantly deteriorated in LH patients. A short-term significant deterioration in VF was also evident in RH patients, which is in accordance with previous research findings.^3^ The most intriguing finding in the RH group was a significant short-term improvement in naming, which, to our knowledge, has not been observed in earlier studies. Over the course of the postoperative period (T2-T3), LH patients showed significant improvement on tests for VF (category and letter) and, to some extent, phonology (sentence repetition). In contrast, RH patients showed improvement only in letter fluency. With regard to the long-term effects of surgery (T1-T3) in RH patients a significant decline was observed for the DIMA sentence completion subtest at the lexico-syntactic level but not on traditional tests. Difficulties with completing more semantically induced sentences may reflect a word-finding deficit, whereas difficulties with less constrained sentences may reflect a syntactic impairment. From a linguistic viewpoint, this makes the sentence completion test the most complex subtest in DIMA. To illustrate, the participant has to understand the sentence fragment, retrieve meaningful words, and organize these words into the correct word order in a fluent manner. Therefore, this test addresses higher linguistic processes that operate not only in language production but also in comprehension, as is seen in natural communication. This finding is in line with an earlier postoperative analysis of spontaneous speech in a group of patients with gliomas, where the number of incomplete sentences increased shortly postoperatively and remained impaired long-term post-surgery.^20^ We observed the aforementioned decline exclusively in RH patients. We speculate that this may be due to a more limited application of intraoperative language tasks (including sentence completion) in cases where preoperative fMRI did not indicate right/-bi-lateralized language function. In contrast, for LH patients, a broader range of language impairments is typically anticipated intraoperatively. Furthermore, in the group of RH patients, we observed a significant decline in DIMA Total, likely reflecting the combined effect of several subtle impairments that, when considered individually, did not reach statistical significance. Similarly to the present study, prior studies also found language deficits in several linguistic domains even though the glioma was located in the RH.^45^ In the LH patients’ group, significant long-term (T1-T3) decline was observed in semantics (semantic odd-picture-out) and partially in phonology (compound word repetition). As mentioned above, only the RH patients’ group showed improvement, specifically on the naming test (BNT).

The findings of this study are in agreement with earlier studies indicating that a complex and distributed architecture in both hemispheres characterizes the neurobiology of language.^46^ Likewise, the results of a long-term postoperative study of spontaneous speech revealed the importance of testing language function regardless of tumor location.^20^ It is plausible that long-term (T1-T3) changes in this study, whether deterioration or recovery, are influenced by different patterns of brain plasticity.^9^ The concept of brain meta-plasticity, i.e. the initial neural activity before synaptic plasticity, was introduced in the field of gliomas to further explain neural changes.^47^ In previous studies, neuroplasticity was found to occur within the tumor, in perilesional regions, and within the affected hemisphere and contralesional hemisphere.^9^ However, we should keep in mind that not all areas have the same potential for neuroplasticity (e.g. ventral premotor cortex, insula, subcortical areas), and that certain types of tumors, the patient’s demographic characteristics, and the time of resection might also affect the extent of neuroplasticity.^9^ We speculate that patients with a delayed decline in our study may have suffered from a glioma located in an area with less potential for neural plasticity. The sample size in our study did not allow for such detailed analyses, as our patient group was too heterogeneous, and we lacked sufficient observations per category.

#### Less vs. more invasive tumors.

—Among the patients with a more invasive tumor in whom we analyzed only the short-term impact of surgery, we observed a T1-T2 decline in phonology (compound and nonword repetition). As mentioned before, a decline in phonology was also observed in the group of patients with less invasive tumors in the LH. Furthermore, a significant effect of hemisphere in Group 2 was observed only in VF (animals), for which patients with right-hemispheric tumors performed better. In this context, it should be noted that patients with more invasive tumors in many cases already started with lower test scores at T1 than patients with less invasive tumors. Hence, a sizeable decline was observed, which failed to reach statistical significance, probably due to the lower sample size in this group.

### Limitations

The first limitation of the study is small sample size at T3, which made it statistically impossible to assess the long-term (T1-T3) effects of awake surgery in Group 2. Secondly, subsequent research could further assess the language impairments of patients by corroborating objectively measured impairments with subjective patient reports. Thirdly, the analysis included only hemispheric location as a variable, but we recognize the importance of considering more specific tumor locations and molecular markers in future research.^9^ Future studies should also elaborate on the question about decline after T3, since this remained open in this study. Next, it is important to note that a statistically significant difference does not necessarily imply clinical relevance. Therefore, the practical implications of our findings should be interpreted with caution. Finally, processing speed was not considered in the present study. Given that previous research highlighted the importance of evaluating response times alongside accuracy, future studies should explore this further.^22,24^

### Clinical Implications and Future Directions

The findings of the present study hold several clinical implications. First and foremost, this study expands the existing knowledge of possible language impairments caused by gliomas located in the nondominant hemisphere and confirms the necessity of comprehensive language testing regardless of the tumor location. The DIMA results show that assessing more complex language abilities at different linguistic levels in patients with gliomas is important, particularly in light of the sentence completion results. This subtest can be especially useful in scenarios where the assessment of spontaneous speech is not feasible and its analysis not always possible. Conversely, a sentence completion subtest, which resembles semi-spontaneous speech, can be analyzed faster and in a standardized way, and can therefore be of high practical value in a clinical setting. We argue that, apart from pre- and postoperative application, this subtest could be used as part of the standard procedure during awake brain surgery regardless of tumor location. For instance, it should not only be used in patients with gliomas in the supplementary motor area in the case of LH tumors, where a reduction of spontaneous speech could typically be present^48^ to overcome long-term deterioration, but also in patients with gliomas in all other areas. Moreover, it is necessary to include tests which assess nonlinguistic processing speed, since delayed responses are a common error in sentence completion tasks (as observed clinically). The current version of DIMA concerns a pen-and-paper test, but a digital DIMA version is currently developed in which reaction times can be measured more adequately.

Additionally, the repetition subtests were also sensitive in capturing language impairments. In fact, DIMA enables the assessment of specific language functions and as such can guide the preparation of language monitoring during awake surgery, as well as the planning of language therapy. Moreover, the naming and VF tests proved to be valuable tools in detecting language impairments related to the tumor or language change after awake surgery.

For patients with more invasive tumors, which typically present more pronounced deficits, we advise to make use of traditional language tests in the pre- and postoperative phase in combination with subtests from DuLIP which was specifically developed for the awake surgery setting^27^ (see suggestion of a minimal test-battery for patients with gliomas in Supplementary Table S5).

To sum up, our results show that mild language impairments in patients with gliomas can persist long-term postsurgery, regardless of severity or grade of the tumor and tumor location. In order not to overlook them and to allow for an objective diagnosis, diagnostic tools aiming to detect mild impairments, such as DIMA, should be used concurrently with the traditional language tests.

## Supplementary material

Supplementary material is available online at *Neuro-Oncology Practice* (https://academic.oup.com/nop/).

npaf090_suppl_Supplementary_Materials_1

## Data Availability

Study protocol, data validation and data analysis plan can be shared upon reasonable request.

## Prior presentations

Preliminary results of this study have been partially presented as posters or oral presentation at the European Association of Neuro-Oncology (EANO) meeting 2021, September 25-26, 2021 (virtual meeting), at the 59th Annual Meeting of the Academy of Aphasia, October 24-26, 2021 (virtual meeting) and at the Science of Aphasia (SoA) 2022, September 12-15, 2022, Bordeaux, France.
