# Supplementary material for: Precision in long-term language evaluation after awake brain tumor surgery
Source: Neurooncol Pract. 2025 Sep 6;13(2):276–91. doi: 10.1093/nop/npaf090 (PMC13153704; doi:10.1093/nop/npaf090)
Supplement: npaf090_suppl_Supplementary_Materials_1 [file npaf090_suppl_supplementary_materials_1.docx]

# Supplementary material

## Supplementary material 1: Methods: DIMA

The DIMA was developed to detect mild aphasia primarily in brain tumor patients ^26^. It was standardized on a representative sample of healthy, adult native Dutch. The DIMA measures language production across different linguistic levels. Phonological subtests evaluate word, compound word, non-word, and sentence repetition abilities. The semantic subtest is an odd-picture-out test requiring patients to name the picture that does not fit with the other two. In the lexico-syntactic subtest, patients complete sentences that vary in semantic predictability: there are semantically induced sentences, which need to be completed with a content word (e.g., 'I wash my hands with...'), and less semantically induced sentences which need to be completed with multiple words or a clause ('At five o’clock...'). The DIMA also includes sentence judgments tests, but these were only administered to a subgroup of patients and are therefore not discussed in the present article ^22^. Apart from the odd-picture-out subtest with five test items, all other subtests contain ten test items, preceded by one or two practice items to familiarize patients with the task. The subtests are time constricted to four seconds. Answers are scored as “incorrect” if they contain errors, slowed responses, hesitations, self-corrections, or repetitions, as specified in the original article ^26^.

## Supplementary Table S2

Number of patients at each time point. Group 1 = astrocytoma, IDH-mutant grade 2 and 3 and oligodendroglioma, IDH-mutant and 1p/19q-codeleted grade 2 and 3; Group 2 = astrocytoma, IDH-mutant grade 4 and glioblastoma, IDH-wildtype.

|  | Group 1 | Group 2 | Total |
| --- | --- | --- | --- |
| T1: baseline testing | 54 | 25 | 79 |
| T2: 3 months postoperatively | 49 | 19 | 68 |
| T3: 1 year postoperatively | 35 | - | 35 |

## Supplementary Table S3

*Percentage of clinical impairments on all language tests.*

|  |  | Tumour type group 1 | | | | Tumour type group 2 | |
| --- | --- | --- | --- | --- | --- | --- | --- |
|  |  | Left hemisphere | | Right hemisphere | |  |  |
|  |  | n | % impaired | n | % impaired | n | % impaired |
| DIMA: Word repetition | T1 | 27 | 0 | 27 | 0 | 25 | 4.0 |
|  | T2 | 23 | 4.3 | 25 | 4.0 | 17 | 23.5 |
|  | T3 | 18 | 0 | 17 | 0 |  |  |
| DIMA: Compound word repetition | T1 | 27 | 7.4 | 27 | 3.7 | 25 | 20.0 |
|  | T2 | 23 | 26.1 | 25 | 8.0 | 17 | 35.3 |
|  | T3 | 18 | 22.2 | 17 | 11.8 |  |  |
| DIMA: Non-word repetition | T1 | 27 | 3.7 | 27 | 3.7 | 25 | 8.0 |
|  | T2 | 23 | 13.0 | 25 | 4.0 | 17 | 23.5 |
|  | T3 | 18 | 5.6 | 17 | 0 |  |  |
| DIMA: Sentence repetition | T1 | 27 | 7.4 | 27 | 11.1 | 25 | 24.0 |
|  | T2 | 23 | 13.0 | 25 | 12.0 | 17 | 35.3 |
|  | T3 | 17 | 5.9 | 17 | 0 |  |  |
| DIMA: Semantic odd-picture-out | T1 | 27 | 3.7 | 26 | 3.8 | 23 | 8.7 |
|  | T2 | 23 | 0 | 25 | 4.0 | 17 | 17.6 |
|  | T3 | 18 | 0 | 17 | 0 |  |  |
| DIMA: Sentence completion | T1 | 27 | 22.2 | 27 | 18.5 | 23 | 30.4 |
|  | T2 | 22 | 4.5 | 25 | 16.0 | 16 | 25.0 |
|  | T3 | 18 | 27.8 | 17 | 29.4 |  |  |
| DIMA: Total | T1 | 27 | 3.7 | 26 | 0 | 21 | 14.3 |
|  | T2 | 22 | 13.6 | 25 | 12.0 | 16 | 25.0 |
|  | T3 | 17 | 5.9 | 17 | 23.5 |  |  |
| Boston Naming Test | T1 | 19 | 47.4 | 21 | 28.6 | 14 | 57.1 |
|  | T2 | 19 | 36.8 | 23 | 39.1 | 15 | 33.3 |
|  | T3 | 16 | 50.0 | 17 | 23.5 |  |  |
| Category Fluency (Animals) | T1 | 26 | 15.4 | 26 | 0 | 22 | 27.3 |
|  | T2 | 24 | 16.7 | 24 | 16.7 | 16 | 18.8 |
|  | T3 | 18 | 11.1 | 17 | 11.8 |  |  |
| Category Fluency (Professions) | T1 | 26 | 19.2 | 26 | 11.5 | 22 | 27.3 |
|  | T2 | 24 | 29.2 | 24 | 29.2 | 16 | 31.2 |
|  | T3 | 17 | 17.6 | 17 | 5.9 |  |  |
| Letter Fluency | T1 | 27 | 11.1 | 26 | 11.5 | 21 | 33.3 |
|  | T2 | 24 | 33.3 | 25 | 20.0 | 15 | 33.3 |
|  | T3 | 18 | 16.7 | 17 | 5.9 |  |  |
| Shortened Token Test | T1 | 27 | 3.7 | 26 | 3.8 | 23 | 21.7 |
|  | T2 | 24 | 4.2 | 23 | 0 | 17 | 23.5 |
|  | T3 | 17 | 0 | 14 | 0 |  |  |

**Supplementary Table S4**

*Outcomes pairwise Fisher’s exact tests, with Benjamini-Hochberg adjustment for multiple testing.*

*p-values and effect size (Cramer’s V). *: p<.05, **; p<.01; ***: p<.001.*

|  |  | Group 1 | | | | | | Group 2 | | |
| --- | --- | --- | --- | --- | --- | --- | --- | --- | --- | --- |
|  |  | Left hemisphere | | | Right hemisphere | | |  |  |  |
|  |  | n | p adj. | V | n | p adj. | V | n | p adj. | V |
| DIMA: Word repetition | T1 vs ctrls | 241 | 1 | .070 | 241 | 1 | .070 | 239 | 1 | .003 |
| DIMA: Compound word repetition | T1 vs ctrls | 240 | .662 | .024 | 240 | 1 | .027 | 238 | .022* | .171 |
| DIMA: Non-word repetition | T1 vs ctrls | 240 | 1 | .015 | 240 | 1 | .015 | 238 | .366 | .046 |
| DIMA: Sentence repetition | T1 vs ctrls | 241 | .533 | .048 | 241 | .208 | .100 | 239 | .002** | .250 |
| DIMA: Semantic odd-picture-out | T1 vs ctrls | 241 | 1 | .001 | 240 | 1 | .002 | 237 | .251 | .073 |
| DIMA: Sentence completion | T1 vs ctrls | 241 | .004** | .235 | 241 | .018* | .193 | 237 | <.001*** | .309 |
| DIMA: Total | T1 vs ctrls | 241 | 1 | .021 | 240 | .614 | .076 | 235 | .118 | .110 |

## Supplementary Table S5

*Suggested minimal language test battery for patients with gliomas*

|  | **Less invasive glioma** | **More invasive glioma** |
| --- | --- | --- |
| *Naming* | BNT | BNT |
| *Fluency* | Letter & category | Letter & category |
| *Phonology/repetition* | DIMA all repetition subtests | DIMA repetition words (singletons), DuLIP (extended) |
| *Semantics* | DIMA odd-picture-out | DuLIP odd-picture-out  DuLIP odd-word out |
| *Syntax* | DIMA sentence completion | DuLIP sentence completion |
| *(Spontaneous speech)* | (semi-/spontaneous speech analysis) | (semi-/spontaneous speech analysis) |
| *Comprehension and the presence and severity of aphasia* |  | shortened Token Test |
